# Cystatin C relates to metabolism in healthy, pubertal adolescents

**DOI:** 10.1007/s00467-021-05209-2

**Published:** 2021-08-25

**Authors:** Niels Ziegelasch, Mandy Vogel, Antje Körner, Eva Koch, Anne Jurkutat, Uta Ceglarek, Katalin Dittrich, Wieland Kiess

**Affiliations:** 1 Hospital for Children and Adolescents, University of Leipzig, Liebigstraße 27b, 04103 Leipzig, Germany; 2 LIFE Leipzig Research Centre for Civilization Diseases, University of Leipzig, Philipp-Rosenthalstrasse 27, 04103 Leipzig, Germany; 3 Centre of Paediatric Research (CPL), University of Leipzig, 04103 Leipzig, Germany; 4 Institute for Laboratory Medicine, Clinical Chemistry and Molecular Diagnostic (ILM), University Hospital Leipzig, 04103 Leipzig, Germany; 5 Present Address: DSO, Walter-Koehn-Str. 1a, Organisationszentrale, D-04356 Leipzig, Germany

**Keywords:** Children, Cystatin C, Puberty, Hormone, Enzyme, Metabolism

## Abstract

**Introduction:**

The cystatin C (CysC) serum level is a marker of glomerular filtration rate and depends on age, gender, and pubertal stage. We hypothesize that CysC might overall reflect energy homeostasis and be regulated by components of the endocrine system and metabolites in pubertal adolescents.

**Methods:**

Serum CysC levels and further possible effector parameters in 5355 fasting, morning venous blood samples from 2035 healthy participants of the LIFE Child cohort study (age 8 to 18 years) were analyzed. Recruitment started in 2011, with probands followed up once a year. Linear univariate and stepwise multivariate regression analyses were performed.

**Results:**

Annual growth rate, serum levels of thyroid hormones, parathyroid hormone, insulin-like growth factor 1, hemoglobin A1c (HbA1c), uric acid, and alkaline phosphatase show relevant and significant associations with CysC serum concentrations (*p* <0.001). Furthermore, male probands’ CysC correlated with the body mass index and testosterone among other sexual hormones. Multivariate analyses revealed that uric acid and HbA1c are associated variables of CysC independent from gender (*p* <0.001). In males, alkaline phosphatase (*p* <0.001) is additionally significantly associated with CysC. Thyroid hormones show significant correlations only in multivariate analyses in females (*p* <0.001).

**Conclusions:**

The described associations strongly suggest an impact of children’s metabolism on CysC serum levels. These alterations need to be considered in kidney diagnostics using CysC in adolescents. Additionally, further studies are needed on CysC in children.

**Graphical abstract:**

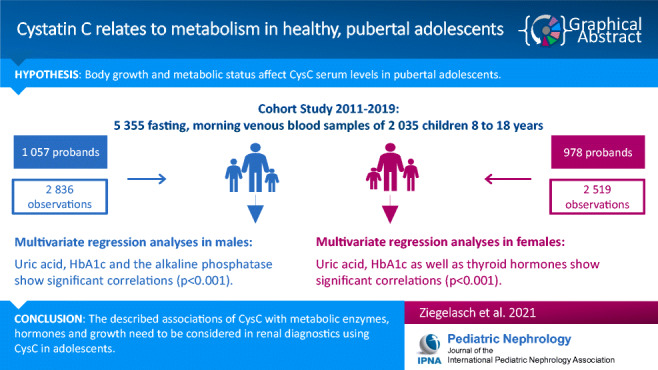

**Supplementary Information:**

The online version contains supplementary material available at 10.1007/s00467-021-05209-2.

## Introduction

Due to its function as an endogenous marker for the glomerular filtration rate (GFR) and thus kidney function, the cysteine protease inhibitor and low molecular weight protein cystatin C (CysC) is of high interest not only in pediatric nephrology [1]. As the product of a housekeeping gene, it is produced by all human nucleated cells at a stable rate [2]. Because it is freely filtered in the glomerulus, it correlates well with GFR determined by gold standard methods, such as the chromium-ethylenediaminetetraacetic acid complex (CrEDTA) or inulin in children and adults [3–6]. Measuring methods include the rapid and precise particle-enhanced turbidimetric and nephelometric immunoassays (PETIA and PENIA) [7, 8]. The studies of Yata et al. (*n* = 1128) and Groesbeck et al. (*n* = 719) first showed that CysC decreases in adolescents aged 15–16 years, being significantly higher in males compared to females at the same age [9, 10]. In our earlier studies, we were able to confirm these findings and proposed the usage of percentiles for the laboratory assessment of CysC in pediatric patients [11]. Whereas serum levels of female adolescents decrease starting at 11 years of age, those for male adolescents increase (ß = 0.028 mg/l/a) until the age of 15 years. From the age of 13 years, they differ significantly from one another [11]. Similar to creatinine, this difference amounts to 15% and is therefore of clinical relevance [10, 11]. Body height and pubertal stage, among others, are good predictors of the CysC serum level [11]. Recently, Miliku et al. suggested that GFR estimated by equations relying on CysC are negatively associated with body mass index (BMI) and body surface area (BSA), but not lean or fat mass percentage [12]. Whereas in adults, the effects of age, gender, height, and weight on CysC are well described [13], they are not satisfactorily assessed in children.

Underlying metabolic and hormonal influences may include blood glucose levels, insulin as well as glucocorticoids [14–16]. Additionally, thyroid dysfunction along with alteration of triiodothyronine (FT3), insulin-like growth factor 1 (IGF-1), and growth hormone (GH) levels are associated with serum CysC levels [17–20]. Further essential impacts on CysC were found in vitro in bone resorption excitatory as well as inhibitory activity [21, 22], uric acid in high school students [23], and serum albumin concentrations in both adults and children [13, 15]. We hypothesize age- and gender-specific correlations of CysC serum concentrations and these indicators of growth and metabolism in generally healthy adolescents [11]. Based on our earlier results, we assume that testosterone and anabolic hormones are significantly associated with CysC serum levels in boys and girls during puberty. Therefore, we aim to investigate potential associations of metabolic enzymes and hormones with serum CysC concentrations in adolescents.

## Material and methods

### Design and study population

Coherence with STROBE (Strengthening the Reporting of Observational Studies in Epidemiology) criteria is shown in annex [24]. The data were assessed within the LIFE Child study in the Leipzig Research Centre for Civilization Diseases (LIFE) [25, 26]. The population-based cohort study is recruiting primarily healthy infants, children, and adolescents in Leipzig (Germany) since 2011. We included measurements of all participants aged 8 to 18 years with valid CysC measurements. Up to 2019, the 2126 participants had one up to eight follow-up visits (once per year). We excluded those with kidney anomalies, nephrolithiasis, or febrile urinary tract infections in their medical history (*n* = 89). The respective diagnoses were obtained by computer-assisted personal interview and sonography diagnostic. Furthermore, we identified and excluded four remaining isolated extreme values of CysC (< 0.4 mg/l), most probably due to measurement errors. Therefore, our study includes 5355 observations of 2035 children in total.

The examinations were performed by trained medical staff following highly standardized procedures [25, 26]. To date, we were able to establish reference intervals for serum CysC levels [11], lipids [27], liver enzymes [28], iron-related blood parameters [29], and metabolites [30] in healthy children. Our study was conducted according to the Declaration of Helsinki and was approved by the Ethics Committee of the University of Leipzig (reg. no. 264-10-19042010), NCT trial number 02550236. All data are pseudomized to comply with the German data protection law and the General Data Protection Regulation of the European Union. More information about our cohort is described by Poulain et al. and Quante et al. [25, 26].

### Laboratory assessment

CysC serum levels and all further laboratory parameters were analyzed in fasting morning venous blood drawn by venipuncture. The Institute for Laboratory Medicine, Clinical Chemistry and Molecular Diagnostic (ILM), University Hospital Leipzig, used an automated laboratory analyzer, Cobas8000 (Roche Diagnostics, Mannheim Germany), following the manufacturer’s protocol. The method used for CysC is the turbidimetric immunoassay (PETIA) Tina-quant® cystatin C (Roche Diagnostics). Primary measurement range is 0.4–8.0 mg/l. Detailed information can be found in Ziegelasch et al. [11]. Furthermore, Suppl. Table 1 summarizes the assessment of the other laboratory parameters along with the respective test, analyzer, and material examined.

### Anthropometric assessment

The measurements of skin plication, weight, and height were taken by our trained staff following highly standardized procedures. The measurement devices, including a stadiometer (measurement accuracy of 0.1 cm) and a Seca 701 scale (measurement accuracy of 50 g), were regularly calibrated. The growth rate was calculated using the difference of height (last visit height subtracted from the current visit height) divided by time in days between two visits in our research center.

### Statistics

Univariate and multivariate analyses were performed using the R software (version 4.0.0) [31]. For multivariate analyses, we used stepwise linear regression analyses (backward deletion) to determine the associations of the independent variables on CysC levels (R-package lme4 [32]). The heatmaps show pairwise correlations between CysC and potentially influencing variables in males and females through illustration of significance levels (*p* values). To account for multiple measurements per subject, the subjects were added as random effect on the intercept in all analyses. Furthermore, all analyses were corrected for age. It is noteworthy that cortisol and uric acid were only assessed in subcohorts with subjects selected by random sampling.

## Results

In total, the data of 2035 participants with 5355 observations was analyzed. The descriptive data are shown in Table 1. Furthermore, the distributions of pubertal stages and BMI in the LIFE Child cohort are summarized in Table 2. Boys enter the pubertal stage II approximately half a year later than girls.

**Table 1:** Descriptive statistics of the LIFE Child cohort (8–18 years)

	Males				Females			
Parameter (unit)	Arithmetic mean	Median	SD	n (obs)	Arithmetic mean	Median	SD	n (obs)
Cystatin C (mg/l)	0.92	0.92	0.12	2836	0.87	0.87	0.11	2519
Growth rate (cm/a)	6.45	6.00	3.81	2156	4.61	4.70	4.13	1950
BMI (SDS)	0.19	0.05	1.17	2830	0.29	0.17	1.23	2505
Skin plication biceps (mm)	7.74	5.80	5.13	2819	9.76	8.07	5.60	2455
Skin plication triceps (mm)	13.28	10.93	7.24	2818	15.82	14.20	6.77	2417
Skin plication iliac crest (mm)	11.86	7.93	9.62	2799	14.31	12.20	8.29	2279
Skin plication subscapular (mm)	10.73	7.60	8.26	2815	11.97	9.20	8.09	2429
Puberty status (Tanner stage)	2.28	2.00	1.43	1767	3.10	3.00	1.52	2197
LH (U/l)	1.99	1.67	1.87	2441	4.37	2.72	6.52	2146
FSH (U/l)	2.64	2.17	1.92	2442	4.30	4.12	2.53	2149
Testosterone (nmol/l)	7.02	1.30	8.42	1415	0.65	0.51	0.86	1298
Estradiol (pmol/l)	40.92	18.40	33.00	1198	201.97	114.70	296.88	1119
TSH (mU/l)	2.63	2.38	2.20	2756	2.44	2.19	1.18	2444
FT3 (pmol/l)	6.57	6.54	0.74	2721	6.21	6.22	0.87	2417
FT4 (pmol/l)	15.73	15.70	2.10	2724	15.50	15.40	2.12	2428
Intact PTH (pmol/l)	3.44	3.26	1.24	2580	3.63	3.44	1.25	2287
Cortisol (nmol/l)	300.65	265.30	145.60	309	323.80	294.70	157.88	283
HbA1c (%)	5.09	5.10	0.30	1726	5.08	5.08	0.34	1641
Glucose (mmol/l)	4.87	4.85	0.42	2723	4.79	4.75	0.59	2423
Insulin (pmol/l)	59.45	51.10	48.80	1235	70.36	62.65	46.45	1200
IGF1 (ng/ml)	245.94	221.20	114.92	2163	281.33	276.90	106.96	1200
Protein, serum (g/l)	71.31	71.40	3.93	2801	71.44	71.40	4.04	1926
Albumin, serum (g/l)	47.67	47.70	2.70	2811	46.95	47.00	2.75	2495
ASAT (μkat/l)	0.50	0.49	0.13	1588	0.45	0.43	0.13	1485
ALAT (μkat/l)	0.35	0.31	0.18	1586	0.30	0.27	0.14	1481
GGT (μkat/l)	0.25	0.22	0.11	1663	0.21	0.19	0.10	1587
ALP (μkat/l)	3.98	3.87	1.42	2792	3.02	3.09	1.48	2487
Uric acid (μmol/l)	301.41	296.50	79.88	632	275.00	274.00	59.97	605
Urea (mmol/l)	4.26	4.20	0.99	1526	3.84	3.80	0.86	1427

Abbreviations: *ALAT* alanine-aminotransferase, alkaline phosphatase, *ASAT* aspartate-aminotransferase, *BMI* body mass index, *CI* confidence interval, *GGT* gamma-glutamyl transferase, *FSH* follicle-stimulating hormone, *FT3* free thyroid hormone 3, *FT4* free thyroid hormone 4, *HbA1c* hemoglobin A1c, *IgG* immunoglobulin G, *IGF1* insulin-like growth factor, *LH* luteinizing hormone, *n* number of observations, *obs* observations, *SD* standard deviation, *PTH* parathyroid hormone, *TSH* thyroid-stimulating hormone

Note: cortisol and uric acid were only assessed in subcohorts with subjects selected by random sampling

**Table 2:** Distribution of puberty status and BMI in the LIFE Child cohort (8–18 years)

	Males			Females		
Pubertal status (Tanner stage)	*n*	Age mean in years	Age SD in years	*n*	Age mean in years	Age SD in years
1	760	9.76	1.20	481	9.35	0.94
2	402	11.63	1.19	392	10.94	1.19
3	170	13.14	1.22	347	12.54	1.30
4	215	14.18	1.25	370	14.15	1.49
5	220	15.73	1.32	607	15.55	1.36
All	1767	12.37	2.61	2197	12.70	2.66
**BMI category**	***n***			***n***		
Underweight	250			196		
Normal	2067			1798		
Overweight	202			188		
Obese	311			323		
All	1479			1406		

Abbreviations: *n* number of observations, *BMI* body mass index

Puberty status: pre-pubertal (Tanner stage 1), pubertal (Tanner stages 2-4), post-pubertal (Tanner stage 5). BMI groups: underweight < 10th percentile, overweight > 90th percentile, obese > 97th percentile

As the puberty status and BMI were not examined in all participants, the total numbers of all observations and participants in this table differ from the numbers presented in Table 3

### Results of univariate linear regression models

**Fig. 1:**
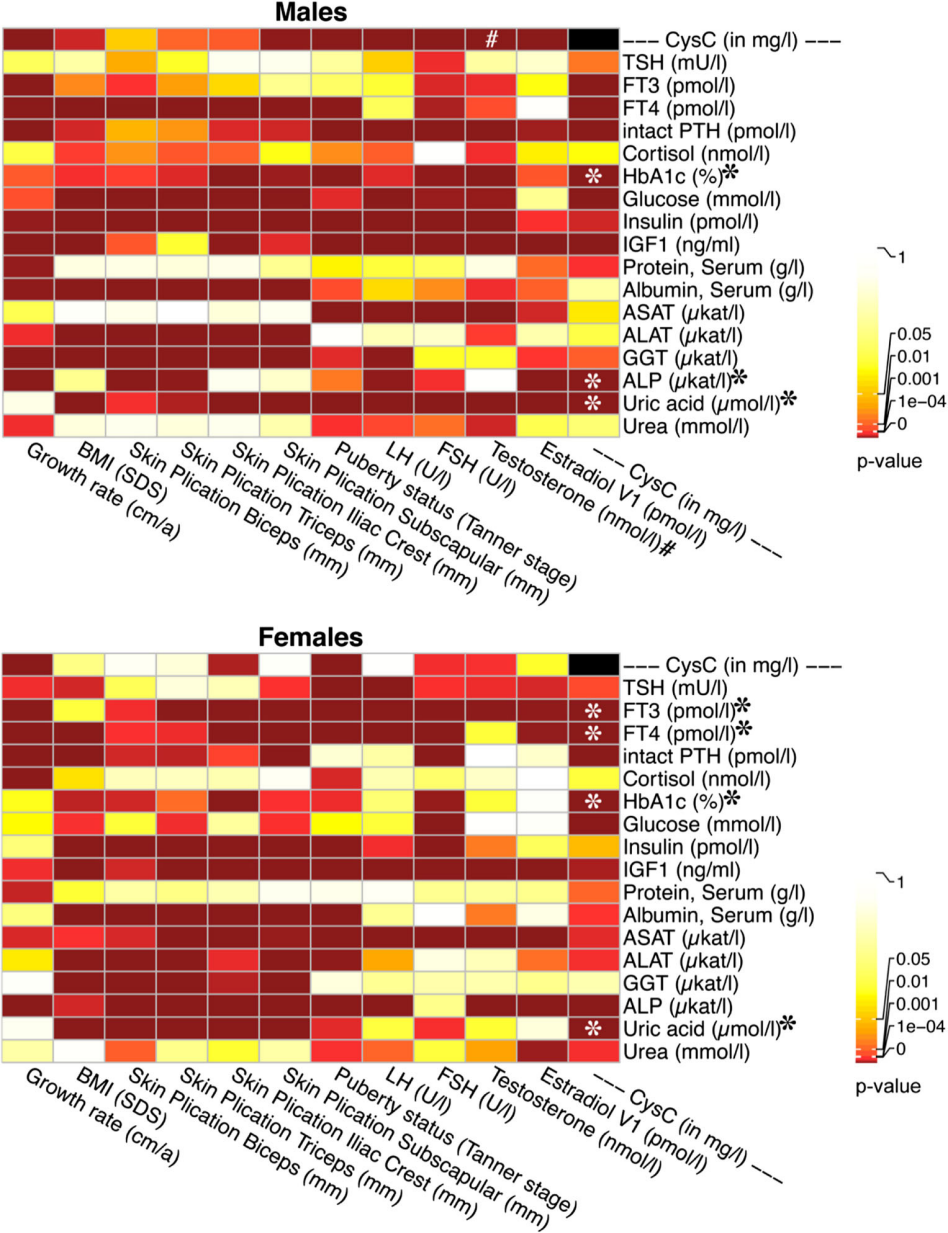
Heatmaps showing pairwise correlations between cystatin C and potentially influencing variables in males and females. Abbreviations: ALAT = alanine-aminotransferase, ALP = alkaline phosphatase, ASAT = aspartate-aminotransferase, BMI = body mass index, CI = confidence interval, GGT = gamma glutamyl transferase, FSH = follicle-stimulating hormone, FT3 = free thyroid hormone 3, FT4 = free thyroid hormone 4, HbA1c = hemoglobin A1c, IgG = immunoglobulin G, IGF1 = insulin-like growth factor, LH = luteinizing hormone, n = number of observations, obs = observations, SD = standard deviation, PTH = parathyroid hormone, TSH = thyroid-stimulating hormone. * marks variables with significant correlations in stepwise multivariate analyses (see also Table 4). # marks testosterone as the strongest potentially influencing, nonsignificant variable in multivariate analyses in male subjects. Anthropometric parameters and sexual hormones are listed in the columns; all other biochemical parameters are shown in the rows. Significance levels (*p* values) are illustrated in colors (0 [brick red], 10^-4^ [red], 0.001 [light red], 0.01 [orange], 0.05 [yellow], 1 [white]). All analyses were corrected for age and repeated measurements in follow-up examination

Table 3 and Fig. 1 show the results of the linear regression models for male and female subjects separately. Sex-independent results of the entire cohort are summarized in Suppl. Table 2. All analyses were corrected for age and repeated measurements per subject by adding the age and pseudonym as random effects.

**Table 3:** Results of univariate linear regression analyses of various parameters with CysC in male and female probands of the LIFE Child Cohort (8–18 years)

	Males								Females							
Parameter (unit*)	ß (mg/l per*)	*p* value	adj. p	Estimate	CI (low)	CI (high)	*R*^2^	obs(n)	ß (mg/l per*)	*p* value	adj. p	Estimate	CI (low)	CI (high)	*R*^2^	obs(n)
Growth rate (cm/a)	0.0076	0.0000	0.0000	0.2177	0.1760	0.2594	0.0476	1774	0.0072	0.0000	0.0000	0.2416	0.1978	0.2855	0.0615	1531
BMI (SDS)	0.0095	0.0001	0.0002	0.0925	0.0474	0.1375	0.0085	2830	0.0028	0.3859	0.5247	0.0305	−0.0183	0.0794	0.0009	2505
Skin plic. biceps (mm)	0.0010	0.0717	0.0946	0.0424	0.0008	0.0840	0.0018	2819	−0.0001	0.9238	0.9882	−0.0061	−0.0506	0.0384	0.0000	2455
Skin plic. triceps (mm)	0.0009	0.0239	0.0329	0.0524	0.0101	0.0947	0.0027	2818	−0.0002	0.7829	0.9507	−0.0103	−0.0552	0.0346	0.0001	2417
Skin plic. iliac crest (mm)	0.0007	0.0206	0.0296	0.0540	0.0119	0.0961	0.0029	2799	−0.0013	0.0001	0.0003	−0.0926	−0.1361	−0.0492	0.0086	2279
Skin plic. subscap. (mm)	0.0015	0.0000	0.0000	0.1001	0.0580	0.1422	0.0100	2815	−0.0001	0.9668	0.9882	−0.0042	−0.0497	0.0413	0.0000	2429
Tanner stage	0.0245	0.0000	0.0000	0.2897	0.2428	0.3367	0.0834	1767	−0.0162	0.0000	0.0000	−0.2196	−0.2625	−0.1767	0.0490	2197
LH (U/l)	0.0220	0.0000	0.0000	0.3396	0.3003	0.3789	0.1129	2441	0.0000	0.9882	0.9882	−0.0025	−0.0402	0.0352	0.0000	2146
FSH (U/l)	0.0180	0.0000	0.0000	0.2851	0.2424	0.3279	0.0790	2442	0.0027	0.0036	0.0097	0.0606	0.0221	0.0990	0.0037	2149
Testosterone (nmol/l)	0.0041	0.0000	0.0000	0.2860	0.2314	0.3406	0.0812	1415	−0.0103	0.0074	0.0180	−0.0762	−0.1279	−0.0245	0.0059	1298
Estradiol (pmol/l)	0.0006	0.0000	0.0000	0.1714	0.1130	0.2298	0.0298	1198	0.0000	0.1375	0.2125	−0.0510	−0.1075	0.0055	0.0027	1119
TSH (mU/l)	0.0002	0.9761	0.9761	0.0032	−0.0345	0.0410	0.0000	2756	0.0051	0.0201	0.0402	0.0528	0.0113	0.0944	0.0028	2444
FT3 (pmol/l)	0.0312	0.0000	0.0000	0.1918	0.1558	0.2277	0.0365	2721	0.0319	0.0000	0.0000	0.2435	0.2058	0.2813	0.0607	2417
FT4 (pmol/l)	−0.0117	0.0000	0.0000	−0.2029	−0.2405	−0.1652	0.0408	2724	−0.0054	0.0000	0.0000	−0.1017	−0.1423	−0.0611	0.0104	2428
Intact PTH (pmol/l)	0.0201	0.0000	0.0000	0.2090	0.1697	0.2483	0.0434	2580	0.0142	0.0000	0.0000	0.1579	0.1169	0.1988	0.0251	2287
Cortisol (nmol/l)	−0.0001	0.1528	0.1801	−0.0920	−0.2048	0.0208	0.0083	309	−0.0001	0.1905	0.2699	−0.0879	−0.2045	0.0286	0.0078	283
HbA1c (%)	−0.1022	0.0000	0.0000	−0.2461	−0.2914	−0.2008	0.0601	1726	−0.0839	0.0000	0.0000	−0.2506	−0.2973	−0.2038	0.0620	1641
Glucose (mmol/l)	0.0319	0.0000	0.0000	0.1118	0.0747	0.1489	0.0125	2723	0.0084	0.0555	0.1048	0.0433	0.0003	0.0863	0.0019	2423
Insulin (pmol/l)	0.0003	0.0000	0.0000	0.1213	0.0656	0.1769	0.0149	1235	−0.0001	0.0962	0.1558	−0.0519	−0.1060	0.0022	0.0027	1200
IGF1 (ng/ml)	0.0510	0.0000	0.0000	0.4510	0.4098	0.4922	0.1906	1860	0.0192	0.0000	0.0000	0.1639	0.1183	0.2095	0.0263	1636
Protein, serum (g/l)	0.0018	0.0043	0.0064	0.0577	0.0185	0.0970	0.0033	2801	0.0011	0.0737	0.1253	0.0378	−0.0030	0.0785	0.0014	2483
Albumin, serum (g/l)	0.0008	0.3442	0.3786	0.0191	−0.0193	0.0575	0.0004	2811	0.0021	0.0127	0.0288	0.0504	0.0112	0.0895	0.0025	2495
ASAT (μkat/l)	0.0429	0.1009	0.1281	0.0438	−0.0053	0.0928	0.0019	1588	0.0808	0.0008	0.0025	0.0893	0.0387	0.1400	0.0080	1485
ALAT (μkat/l)	0.0269	0.1515	0.1801	0.0402	−0.0082	0.0886	0.0016	1586	0.0567	0.0183	0.0389	0.0664	0.0146	0.1183	0.0045	1481
GGT (μkat/l)	0.0858	0.0039	0.0061	0.0763	0.0270	0.1257	0.0058	1663	0.0058	0.9558	0.9882	0.0053	−0.0502	0.0608	0.0000	1587
ALP (μkat/l)	0.0218	0.0000	0.0000	0.2606	0.2242	0.2970	0.0664	2792	0.0246	0.0000	0.0000	0.3230	0.2846	0.3615	0.1035	2487
Uric acid (μmol/l)	0.0006	0.0000	0.0000	0.4210	0.3398	0.5022	0.1666	632	0.0005	0.0000	0.0000	0.2510	0.1605	0.3416	0.0626	605
Urea (mmol/l)	−0.0030	0.3978	0.4235	−0.0243	−0.0742	0.0256	0.0006	1526	0.0109	0.0037	0.0097	0.0798	0.0275	0.1322	0.0065	1427

Abbreviations: *adj. p* adjusted *p* value, *ALAT* alanine-aminotransferase, *ALP* alkaline phosphatase, *ASAT* aspartate-aminotransferase, *BMI* body mass index, *CI* confidence interval, *GGT* gamma glutamyl transferase, *FSH* follicle-stimulating hormone, *FT3* free thyroid hormone 3, *FT4* free thyroid hormone 4, *HbA1c* hemoglobin A1c, *IgG* immunoglobulin G, *IGF1* insulin-like growth factor, *LH* luteinizing hormone, *n* number of observations, *obs* observations, *Plic.* plication, *R*^2^ explained variation of the final model, *SD* standard deviation, *Subscap*. subscapular, *PTH* parathyroid hormone, *TSH* thyroid-stimulating hormone. All analyses were corrected for age and multiple testing in follow-up examinations

Note: cortisol and uric acid were only assessed in subcohorts with subjects selected by random sampling

In the entire study population, CysC is positively correlated with the growth rate as well as BMI (*p* <0.01). This effect can also be seen in the male subcohort. Additionally, in males, the puberty status (*p* <0.001), the iliac, triceps (*p* <0.05), and the subscapular skin folds in millimeters (*p* <0.001) are positively associated with CysC serum levels. Nevertheless, in the female subcohort, only growth rate, iliac skin plication, and pubertal status show significant associations (*p* <0.001).

Regarding the endocrine system, testosterone is positively and significantly associated with CysC in males (*p* <0.001), but negatively in females (*p* <0.01). Furthermore, in males, follicle-stimulating hormone (FSH) and luteinizing hormone (LH) correlate positively with serum concentrations of CysC (*p* <0.001), but with only a small effect size for LH. In females, FSH shows similar significant correlations with CysC, whereas LH and estradiol have only very small effect sizes. Of all thyroid hormones, FT3 and free thyroid hormone 4 (FT4) show inverse correlations with CysC (*p* <0.001). Besides, parathyroid hormone (PTH) is associated with serum CysC levels independently from gender, whereas no association with cortisol could be found. IGF1 and in males insulin are two further hormones that show significant correlations with CysC (*p* <0.001).

Metabolic enzymes and further parameters were investigated. Hemoglobin A1c (HbA1c) and in male probands the blood glucose level also correlate significantly with CysC serum concentrations (*p* <0.001). An association can also be seen with serum protein and gamma-glutamyl transferase in males as well as with albumin, urea, alanine-aminotransferase (ALAT, *p* <0.05), and aspartate-aminotransferase serum levels (ASAT, *p* <0.001) in female probands. Finally, uric acid and alkaline phosphatase are associated with serum CysC independent from gender (*p* <0.001).

### Results of hierarchical multivariate regression models

In stepwise multivariate linear regression analysis (backward deletion), we determined the factors having the most significant statistical association and the highest effect size on CysC. All factors showing statistically significant effects on CysC in univariate linear regression models including anthropometric data were considered in the analyses. The results are shown in Table 4.

**Table 4:** Results of stepwise multivariate analyses of various parameters with CysC in males and females

Males					Females				
Parameter (unit*)	ß (mg/l per*)	std. error	*z* value	*p* value	Parameter (unit*)	ß (mg/l per*)	std. error	*z* value	*p* value
Testosterone (nmol/l)	0.0021	0.0008	2.513	0.0505	FT3 (pmol/l)	0.0300	0.0051	5.894	< 0.001
ALP (μkat/l)	0.0165	0.0044	3.741	< 0.001	FT4 (pmol/l)	−0.0117	0.0022	−5.236	< 0.001
HBA1C (%)	−0.1107	0.0170	−6.528	< 0.001	HBA1C (%)	−0.1026	0.0133	−7.711	< 0.001
Uric acid (μmol/l)	0.0005	0.0001	6.315	< 0.001	Uric acid (μmol/l)	0.0005	0.0001	5.931	< 0.001

*ALP* aspartate-aminotransferase, *FT3* free thyroid hormone 3, *FT4* free thyroid hormone 4, *HbA1c* hemoglobin A1c

The analysis includes 360 and 516 probands for males (*R*^2^ = 0.26) and females (*R*^2^ = 0.26), respectively. Independent variables included in this stepwise multivariate analyses: growth rate (cm/a), BMI (SDS), skin plication Iliac crest (mm, only in females’ analysis), skin plication subscapular (mm, only in males’ analysis), puberty status (Tanner stage), LH (U/l), FSH (U/l), testosterone (nmol/l), estradiol (pmol/l), TSH (mU/l), FT3 (pmol/l), FT4 (pmol/l), intact PTH (pmol/l), cortisol (nmol/l), HbA1c (%), glucose (mmol/l), insulin (pmol/l), IGF1 (ng/ml), protein (g/l), albumin (g/l), ASAT (μkat/l), ALAT (μkat/l), GGT (μkat/l), ALP (μkat/l), uric acid (μmol/l), urea (mmol/l)

The final model for females includes the free thyroid hormones FT3 and FT4 (*p* <0.001) that are both inversely associated with CysC serum concentrations with *ß* = 0.0300 and *ß* = −0.0117, respectively. In addition, HbA1c (*ß* = −0.1026) and uric acid (*ß* = 0.5 × 10^−3^) show significant correlations (*p* <0.001).

In male participants, the last four remaining effector variables after stepwise deletion were the alkaline phosphatase (*ß* = 0.0165, *p* <0.001), HbA1c (*ß* = −0.1107, *p* <0.001), uric acid (*ß* = 0.0005, *p* <0.001), and testosterone (*ß* = 0.0021, *p* = 0.051).

## Discussion

CysC levels were shown to be associated with height, age, and puberty [11], with puberty status being a strong predictor of CysC serum concentrations, especially in 11- to 14-year-old adolescents. This is coherent with previous studies [9, 10, 33]. Likewise, we found a positive correlation of pubertal status with CysC in boys and a negative correlation in girls. The growth rate is one strong predictor of serum CysC levels in all participants in univariate analyses. However, in the multivariate regression model, it does not exceed the effect of the factors described in Table 4. In contrast to girls, boys’ serum CysC concentrations are associated with the BMI. The effect of BMI has earlier been investigated by Alosco et al. before and 12 months after bariatric surgery, showing that BMI as well as CysC significantly decreased [34]. Another large study with more than 8000 adults showed that age, gender, weight, and height were associated with CysC levels [13]. Marmarinos et al. described an interaction of CysC and BMI. However, Miliku et al. found no correlation with the lean or fat mass percentage in 6-year-old children, but did not investigate children or adolescents during puberty [12, 33]. Overall, we assume an effect of body composition and development on CysC due to altering metabolism. This effect is most obvious in pubertal boys and may be explained by a higher growth rate compared to adolescent girls with an anabolic status.

Underlying hormonal mechanisms are already discussed in earlier studies: GH and FT3 enhance the production of CysC in adipocytes [18]. In vivo, FT3 enhances CysC levels in patients with hyperthyroidism [19]. When examining post-transsphenoidal surgery patients, CysC declined similarly to GH and IGF1 [20]. A positive correlation of FT3 is confirmed in our study, whereas FT4 is negatively associated with CysC. These two parameters are showing significant, but inverse associations with CysC serum concentrations in multivariate analyses in girls. The deiodinase may explain this inverse effect, although, to our knowledge, no studies investigating this potential association exist to date. Only in patients with thyroid dysfunction, CysC levels were already described to moderately but significantly rise along with thyroxin blood levels [17]. IGF-1 is another factor with relevant effect in both males and females, which, however, does not exceed the effect of the factors in the described multivariate analyses. One similarity of all these potential effector variables is their physiological function in the body’s metabolism and body growth.

After stepwise deletion in the multivariate analyses, testosterone was among the four remaining factors with the strongest correlations to serum CysC with a final *ß* of 0.505 (*p* = 0.051). To our knowledge, no studies examining this correlation to CysC in children exist so far. Veldhuis et al. only found lower estradiol and increased testosterone associated with higher IGF-I in healthy aging adults, suggesting IGF-1 as a potential mediator variable [35]. Nevertheless, Phillip et al. did not find any effects of testosterone on CysC when investigating hypophysectomized castrated rats [36]. Therefore, an association of testosterone and CysC may depend on the pituitary gland activity, maybe through activation of the GH axis.

In our study, the anabolic hormone insulin showed a significant correlation to CysC only in males. Therefore, insulin may be another potential mediator or effector in the interdependence of CysC and pubertal stage in boys. Testosterone induces an anabolic metabolism that may result in an increasing insulin serum level. Meanwhile, no relevant effect of the metabolic hormone cortisol on CysC could be found. So far, only studies in children treated with glucocorticoids for malignancy or kidney disease were proven to have elevated levels of serum CysC [14–16]. An interaction of glucocorticoid levels and CysC is not described in healthy children yet.

Finally, we examined the association of indicators of cell and body metabolism with CysC. Lerner and Grubb described a positive correlation of CysC with bone resorption as well as its potential function of antagonizing the effect of PTH and PTH-related peptide on bone metabolism [22]. The accumulation of CysC in milk basic protein in vitro was positively correlated to osteoblastic proliferation as well as bone resorption inhibitory activity in a study by Yasueda et al. [21], confirming the findings of Lerner and Grubb. In our study, PTH was associated with CysC with *ß* = 0.017 (*p* <0.001) independent from gender. On the contrary, alkaline phosphatase as an indicator of bone metabolism remained one potential effector variable of CysC in multivariate analyses in boys, not in girls. Nevertheless, the effect does not differ between boys and girls in univariate analyses (*ß*_boys_ = 0.022 and *ß*_girls_ = 0.025, both *p* <0.001). We assume that alkaline phosphatase may increase especially in pubertal boys with an elevated growth rate compared to girls. Other potential indicators of cell metabolism, i.e., cellular processes of degradation and new formation, include uric acid, whose direct correlation with CysC was shown to be higher in boys than girls [23]. Likewise, serum albumin concentrations are known to be associated with lower concentrations of CysC [13, 15]. We found uric acid to be a strong predictor of serum CysC levels in both males and females, even in multivariate analyses (*ß* = 0.005, *p* <0.001), whereas serum albumin concentrations showed no relevant impact on CysC. However, uric acid was only assessed in subcohorts with subjects selected by random sampling (*n* = 1237). The high correlation with CysC in multivariate analyses may be explained by the physiological role of uric acid in cell metabolism as mentioned above. Additionally, uric acid is renally excreted and therefore depends on kidney function with CysC as an important indicator.

The metabolic function of the liver may also alter CysC serum concentrations: associations with the matrix metalloproteinase 2 and hepatic diseases both positively correlated with CysC [37]. Furthermore, a direct correlation with liver fibrosis stages and the severity of liver diseases was found [38–40]. However, associations of liver enzymes and CysC in primarily healthy subjects were not described yet. We found only low associations with ASAT and ALAT, as well as urea in girls, with gamma-glutamyl transferase in boys. They may indicate increased metabolic activity in pubertal adolescents, although this potential interdependence would not explain the differences between boys and girls found in this study.

Limitations of this study include the methodology: CysC concentrations were assessed by turbidimetric assays, whereas a previous meta-analysis favors nephelometric assays [41]. The different methodology may explain discrepancies of our results with those of other studies. Furthermore, correlation does not necessarily mean a causal relationship between cystatin C and the considered variable. Causality may for example be determined by Mendelian randomization studies. Besides, the homogeneity of our primarily Caucasian cohort of Leipzig limits the generalizability of the results.

Overall, we assume that body growth in boys due to pubertal development affects the synthesis of the housekeeping protein CysC. Its positive correlation with testosterone and alkaline phosphatase as predictors of body growth underline this thesis. The underlying mechanisms may include the hormonal stimulation of the body’s cell metabolism. This may also explain the high accuracy of eGFR equations such as the Andersen formula including the body cell mass [42, 43].

## Conclusions

The results show that differences in CysC during puberty are associated with testosterone and other indicators of cell metabolism and growth. Due to the broad age range from 8 to 18 years, a large number of observations (*n* = 5335) of healthy participants, and a standardized assessment, the results are of high accuracy. They emphasize the necessity of using percentiles of CysC serum levels in adolescents in kidney diagnostics. Additionally, further studies are needed on CysC in children.

## Supplementary information

ESM 1 (DOCX 22 kb)

ESM 2 A higher resolution version of the Graphical abstract is available as Supplementary information (PPTX 43.8 kb)

## Data Availability

All data generated or analyzed during this study are included in this published article and its supplementary information files.

## Code availability

The R scripts are available in this article’s supplementary information files.

## References

[1] Brzin J, Popovic T, Turk V, Borchart U, Machleidt W (1984). Human cystatin, a new protein inhibitor of cysteine proteinases. Biochem Biophys Res Commun.

[2] Abrahamson M, Olafsson I, Palsdottir A, Ulvsback M, Lundwall A, Jensson O (1990). Structure and expression of the human cystatin C gene. Biochem J.

[3] Ylinen EA, Ala-Houhala M, Harmoinen AP, Knip M (1999). Cystatin C as a marker for glomerular filtration rate in pediatric patients. Pediatr Nephrol.

[4] Grubb A, Simonsen O, Sturfelt G, Truedsson L, Thysell H (1985). Serum concentration of cystatin C, factor D and beta 2-microglobulin as a measure of glomerular filtration rate. Acta Med Scand.

[5] Tenstad O, Roald AB, Grubb A, Aukland K (1996). Renal handling of radiolabelled human cystatin C in the rat. Scand J Clin Lab Invest.

[6] Filler G, Bokenkamp A, Hofmann W, Le Bricon T, Martinez-Bru C, Grubb A (2005). Cystatin C as a marker of GFR--history, indications, and future research. Clin Biochem.

[7] Kyhse-Andersen J, Schmidt C, Nordin G, Andersson B, Nilsson-Ehle P, Lindstrom V (1994). Serum cystatin C, determined by a rapid, automated particle-enhanced turbidimetric method, is a better marker than serum creatinine for glomerular filtration rate. Clin Chem.

[8] Mussap M, Ruzzante N, Varagnolo M, Plebani M (1998). Quantitative automated particle-enhanced immunonephelometric assay for the routinary measurement of human cystatin C. Clin Chem Lab Med.

[9] Yata N, Uemura O, Honda M, Matsuyama T, Ishikura K, Hataya H (2013). Reference ranges for serum cystatin C measurements in Japanese children by using 4 automated assays. Clin Exp Nephrol.

[10] Groesbeck D, Kottgen A, Parekh R, Selvin E, Schwartz GJ, Coresh J (2008). Age, gender, and race effects on cystatin C levels in US adolescents. Clin J Am Soc Nephrol.

[11] Ziegelasch N, Vogel M, Muller E, Tremel N, Jurkutat A, Loffler M (2019). Cystatin C serum levels in healthy children are related to age, gender, and pubertal stage. Pediatr Nephrol.

[12] Miliku K, Bakker H, Dorresteijn EM, Cransberg K, Franco OH, Felix JF (2017). Childhood estimates of glomerular filtration rate based on creatinine and cystatin C: importance of body composition. Am J Nephrol.

[13] Knight EL, Verhave JC, Spiegelman D, Hillege HL, de Zeeuw D, Curhan GC (2004). Factors influencing serum cystatin C levels other than renal function and the impact on renal function measurement. Kidney Int.

[14] Stevens LA, Schmid CH, Greene T, Li L, Beck GJ, Joffe MM (2009). Factors other than glomerular filtration rate affect serum cystatin C levels. Kidney Int.

[15] Bokenkamp A, Laarman CARC, Braam KI, van Wijk JAE, Kors WA, Kool M (2007). Effect of corticosteroid therapy on low-molecular weight protein markers of kidney function. Clin Chem.

[16] Risch L, Herklotz R, Blumberg A, Huber AR (2001). Effects of glucocorticoid immunosuppression on serum cystatin C concentrations in renal transplant patients. Clin Chem.

[17] Wiesli P, Schwegler B, Spinas GA, Schmid C (2003). Serum cystatin C is sensitive to small changes in thyroid function. Clin Chim Acta Int J Clin Chem.

[18] Schmid C, Ghirlanda C, Zwimpfer C, Tschopp O, Zuellig RA, Niessen M (2019). Cystatin C in adipose tissue and stimulation of its production by growth hormone and triiodothyronine in 3T3-L1 cells. Mol Cell Endocrinol.

[19] Kotajima N, Yanagawa Y, Aoki T, Tsunekawa K, Morimura T, Ogiwara T (2010). Influence of thyroid hormones and transforming growth factor-beta1 on cystatin C concentrations. J Int Med Res.

[20] Sze L, Bernays RL, Zwimpfer C, Wiesli P, Brandle M, Schmid C (2013). Impact of Growth Hormone on Cystatin C. Nephron Extra.

[21] Yasueda T, Abe Y, Shiba M, Kamo Y, Seto Y (2018). A new insight into cystatin C contained in milk basic protein to bone metabolism: effects on osteoclasts and osteoblastic MC3T3-E1 cells in vitro. Anim Sci J Nihon Chikusan Gakkaiho.

[22] Lerner UH, Grubb A (1992). Human cystatin C, a cysteine proteinase inhibitor, inhibits bone resorption in vitro stimulated by parathyroid hormone and parathyroid hormone-related peptide of malignancy. J Bone Miner Res.

[23] Harada M, Izawa A, Hidaka H, Nakanishi K, Terasawa F, Motoki H (2017). Importance of cystatin C and uric acid levels in the association of cardiometabolic risk factors in Japanese junior high school students. J Cardiol.

[24] Vandenbroucke JP, von Elm E, Altman DG, Gotzsche PC, Mulrow CD, Pocock SJ (2007). Strengthening the Reporting of Observational Studies in Epidemiology (STROBE): explanation and elaboration. Ann Intern Med.

[25] Poulain T, Baber R, Vogel M, Pietzner D, Kirsten T, Jurkutat A (2017). The LIFE Child study: a population-based perinatal and pediatric cohort in Germany. Eur J Epidemiol.

[26] Quante M, Hesse M, Dohnert M, Fuchs M, Hirsch C, Sergeyev E (2012). The LIFE child study: a life course approach to disease and health. BMC Public Health.

[27] Dathan-Stumpf A, Vogel M, Hiemisch A, Thiery J, Burkhardt R, Kratzsch J (2016). Pediatric reference data of serum lipids and prevalence of dyslipidemia: Results from a population-based cohort in Germany. Clin Biochem.

[28] Bussler S, Vogel M, Pietzner D, Harms K, Buzek T, Penke M (2018). New pediatric percentiles of liver enzyme serum levels (alanine aminotransferase, aspartate aminotransferase, γ-glutamyltransferase): Effects of age, sex, body mass index, and pubertal stage. Hepatology.

[29] Rieger K, Vogel M, Engel C, Ceglarek U, Harms K, Wurst U (2018). Does physiological distribution of blood parameters in children depend on socioeconomic status? Results of a German cross-sectional study. BMJ Open.

[30] Hirschel J, Vogel M, Baber R, Garten A, Beuchel C-F, Dietz Y (2020). Relation of Whole Blood Amino Acid and 3 Acylcarnitine Metabolome to Age, Sex, BMI, Puberty, 4 and Metabolic Markers in Children and Adolescents. Metabolites.

[31] Core Team R (2016). R: A language and environment for statistical computing.

[32] Bates D, Maechler M, Bolker B, Walker S (2015). Fitting Linear Mixed-Effects Models Using lme4. J Stat Softw.

[33] Marmarinos A, Garoufi A, Panagoulia A, Dimou S, Drakatos A, Paraskakis I (2016). Cystatin-C levels in healthy children and adolescents: Influence of age, gender, body mass index and blood pressure. Clin Biochem.

[34] Alosco ML, Spitznagel MB, Strain G, Devlin M, Cohen R, Crosby RD (2014). The effects of cystatin C and alkaline phosphatase changes on cognitive function. J Neurol Sci.

[35] Veldhuis JD, Frystyk J, Iranmanesh A, Orskov H (2005). Testosterone and estradiol regulate free insulin-like growth factor I (IGF-I), IGF binding protein 1 (IGFBP-1), and dimeric IGF-I/IGFBP-1 concentrations. J Clin Endocrinol Metab.

[36] Phillip M, Palese T, Hernandez ER, Roberts CTJ, LeRoith D, Kowarski AA (1992). Effect of testosterone on insulin-like growth factor-I (IGF-I) and IGF-I receptor gene expression in the hypophysectomized rat. Endocrinology.

[37] Chen T-Y, Hsieh Y-S, Yang C-C, Wang C-P, Yang S-F, Cheng Y-W (2005). Relationship between matrix metalloproteinase-2 activity and cystatin C levels in patients with hepatic disease. Clin Biochem.

[38] Takeuchi M, Fukuda Y, Nakano I, Katano Y, Hayakawa T (2001). Elevation of serum cystatin C concentrations in patients with chronic liver disease. Eur J Gastroenterol Hepatol.

[39] Chu S-C, Wang C-P, Chang Y-H, Hsieh Y-S, Yang S-F, Su J-M (2004). Increased cystatin C serum concentrations in patients with hepatic diseases of various severities. Clin Chim Acta.

[40] Ladero JM, Cardenas MC, Ortega L, Gonzalez-Pino A, Cuenca F, Morales C (2012). Serum cystatin C: a non-invasive marker of liver fibrosis or of current liver fibrogenesis in chronic hepatitis C?. Ann Hepatol.

[41] Wei L, Ye X, Pei X, Wu J, Zhao W (2015). Diagnostic accuracy of serum cystatin C in chronic kidney disease: a meta-analysis. Clin Nephrol.

[42] Onerli Salman D, Siklar Z, Cullas Ilarslan EN, Ozcakar ZB, Kocaay P, Berberoglu M (2019). Evaluation of Renal Function in Obese Children and Adolescents Using Serum Cystatin C Levels, Estimated Glomerular Filtration Rate Formulae and Proteinuria: Which is most Useful?. J Clin Res Pediatr Endocrinol.

[43] Bjork J, Nyman U, Berg U, Delanaye P, Dubourg L, Goffin K (2019). Validation of standardized creatinine and cystatin C GFR estimating equations in a large multicentre European cohort of children. Pediatr Nephrol.

