# Supplementary material for: Cystatin C relates to metabolism in healthy, pubertal adolescents
Source: Pediatr Nephrol. 2021 Aug 25;37(2):423–32. doi: 10.1007/s00467-021-05209-2 (PMC8816513; doi:10.1007/s00467-021-05209-2)
Supplement: Supplementary file 1 — (DOCX 22 kb) [file 467_2021_5209_MOESM1_ESM.docx]

Suppl. Table 1. Overview of methods of the laboratory assessments

| **Parameter** | **Test** | **Analyzer** | **Material** |
| --- | --- | --- | --- |
| CysC (mg/l) | Turbidimetric immunoassay (PETIA) | Roche Cobas | Serum |
| LH (U/l) | Electro-chemiluminescence immunoassay | Roche Cobas | Serum |
| FSH (U/l) | Electro-chemiluminescence immunoassay | Roche Cobas | Serum |
| Testosterone (nmol/l) | Electro-chemiluminescence immunoassay | Roche Cobas | Serum |
| Estradiol (pmol/l) | Electro-chemiluminescence immunoassay | Roche Cobas | Serum |
| TSH (mU/l) | Electro-chemiluminescence immunoassay | Roche Cobas | Serum |
| FT3 (pmol/l) | Electro-chemiluminescence immunoassay | Roche Cobas | Serum |
| FT4 (pmol/l) | Electro-chemiluminescence immunoassay | Roche Cobas | Serum |
| intaktes PTH (pmol/l) | Electro-chemiluminescence immunoassay | Roche Cobas | Serum |
| Cortisol (nmol/l) | Electro-chemiluminescence immunoassay | Roche Cobas | Serum |
| HbA1c (%) | Turbidimetry | Roche Cobas | EDTA |
| Glucose (mmol/l) | UV photometric method | Roche Cobas | Serum |
| Insulin (pmol/l) | Electro-chemiluminescence immunoassay | Roche Cobas | Serum |
| IGF1 (ng/ml) | Chemiluminescence immunoassay | IDS iSYS | Serum |
| Protein, serum (g/l) | Photometric colorimetry | Roche Cobas | Serum |
| Albumin, serum (g/l) | Photometric colorimetry | Roche Cobas | Serum |
| ASAT (µkat/l) | UV photometric method | Roche Cobas | Serum |
| ALAT (µkat/l) | UV photometric method | Roche Cobas | Serum |
| GGT (µkat/l) | Enzymatic colorimetry | Roche Cobas | Serum |
| ALP (µkat/l) | Colorimetry | Roche Cobas | Serum |
| Uric Acid(µmol/l) | Enzymatic colorimetry | Roche Cobas | Serum |
| Urea (mmol/l) | Kinetic test | Roche Cobas | Serum |

List of parameters assessed in our Institute for Laboratory Medicine, Clinical Chemistry and Molecular Diagnostic (ILM) with the according test, analyzer, and material examined

Suppl. Table 2. Results of univariate analyses in all probands of the LIFE Child cohort (8–18 years)

| **Parameter (unit*)** | **ß (mg/l per*)** | ***p* value** | **adj. p** | **Estimate** | **CI (low)** | **CI (high)** | **obs(n)** |
| --- | --- | --- | --- | --- | --- | --- | --- |
| Growth rate (cm/a) | 0.0083 | 0.0000 | 0.0000 | 0.2547 | 0.2247 | 0.2847 | 3305 |
| BMI (SDS) | 0.0059 | 0.0010 | 0.0019 | 0.0594 | 0.0260 | 0.0928 | 5335 |
| Skin plication biceps (mm) | −0.0001 | 0.8553 | 0.9088 | −0.0061 | −0.0367 | 0.0245 | 5274 |
| Skin plication triceps (mm) | 0.0000 | 0.9862 | 0.9862 | −0.0017 | −0.0327 | 0.0294 | 5235 |
| Skin plication iliac crest (mm) | −0.0003 | 0.1468 | 0.1920 | −0.0261 | −0.0568 | 0.0045 | 5078 |
| Skin plication subscapular (mm) | 0.0007 | 0.0070 | 0.0108 | 0.0456 | 0.0145 | 0.0767 | 5244 |
| Puberty status (Tanner stage) | −0.0013 | 0.4500 | 0.5100 | −0.0165 | −0.0496 | 0.0167 | 3964 |
| LH (U/l) | 0.0008 | 0.0493 | 0.0670 | 0.0307 | 0.0037 | 0.0577 | 4587 |
| FSH (U/l) | 0.0054 | 0.0000 | 0.0000 | 0.1061 | 0.0768 | 0.1355 | 4591 |
| Testosterone (nmol/l) | 0.0046 | 0.0000 | 0.0000 | 0.2625 | 0.2224 | 0.3026 | 2713 |
| Estradiol (pmol/l) | 0.0000 | 0.0004 | 0.0008 | −0.0754 | −0.1150 | −0.0358 | 2317 |
| TSH (mU/l) | 0.0015 | 0.1702 | 0.2143 | 0.0228 | −0.0050 | 0.0507 | 5200 |
| FT3 (pmol/l) | 0.0349 | 0.0000 | 0.0000 | 0.2389 | 0.2129 | 0.2649 | 5138 |
| FT4 (pmol/l) | −0.0086 | 0.0000 | 0.0000 | −0.1519 | −0.1793 | −0.1245 | 5152 |
| Intact PTH (pmol/l) | 0.0169 | 0.0000 | 0.0000 | 0.1776 | 0.1492 | 0.2059 | 4867 |
| Cortisol (nmol/l) | −0.0001 | 0.0329 | 0.0466 | −0.0947 | −0.1758 | −0.0137 | 592 |
| HbA1c (%) | −0.0919 | 0.0000 | 0.0000 | −0.2415 | −0.2740 | −0.2091 | 3367 |
| Glucose (mmol/l) | 0.0208 | 0.0000 | 0.0000 | 0.0883 | 0.0598 | 0.1167 | 5146 |
| Insulin (pmol/l) | 0.0001 | 0.3352 | 0.3930 | 0.0242 | −0.0148 | 0.0632 | 2435 |
| IGF1 (ng/ml) | 0.0337 | 0.0000 | 0.0000 | 0.3038 | 0.2721 | 0.3355 | 3496 |
| Protein, serum (g/l) | 0.0013 | 0.0023 | 0.0039 | 0.0440 | 0.0159 | 0.0721 | 5284 |
| Albumin, serum (g/l) | 0.0019 | 0.0015 | 0.0027 | 0.0446 | 0.0173 | 0.0719 | 5306 |
| ASAT (µkat/l) | 0.0870 | 0.0000 | 0.0000 | 0.0922 | 0.0572 | 0.1272 | 3073 |
| ALAT (µkat/l) | 0.0525 | 0.0002 | 0.0004 | 0.0705 | 0.0351 | 0.1059 | 3067 |
| GGT (µkat/l) | 0.0877 | 0.0001 | 0.0002 | 0.0779 | 0.0407 | 0.1151 | 3250 |
| ALP (µkat/l) | 0.0253 | 0.0000 | 0.0000 | 0.3248 | 0.2982 | 0.3513 | 5279 |
| Uric acid (µmol/l) | 0.0006 | 0.0000 | 0.0000 | 0.3757 | 0.3162 | 0.4352 | 1237 |
| Urea (mmol/l) | 0.0069 | 0.0053 | 0.0086 | 0.0533 | 0.0170 | 0.0896 | 2953 |

Abbreviations: adj. p adjusted p value, ALAT alanine-aminotransferase, ALP alkaline phosphatase, ASAT aspartate-aminotransferase, BMI body mass index, CI confidence interval, GGT gamma-glutamyl transferase, FSH follicle-stimulating hormone, FT3 free thyroid hormone 3, FT4 free thyroid hormone 4, HbA1c hemoglobin A1c, IgG immunoglobulin G, IGF1 insulin-like growth factor, LH luteinizing hormone, n number of observations, obs observations, SD standard deviation, PTH parathyroid hormone, TSH thyroid-stimulating hormone

All analyses were corrected for age and multiple testing in follow-up examinations. Note: cortisol and uric acid were only assessed in subcohorts with subjects selected by random sampling

Suppl. File 1. Original data of the LIFE Child cohort (8–18 years) including all probands from 2011–2019

Suppl. File 2. R script for descriptive, univariate, and multivariate analyses
