# Supplementary material for: Cystatin C relates to metabolism in healthy, pubertal adolescents
Source: Pediatr Nephrol. 2021 Aug 25;37(2):423–32. doi: 10.1007/s00467-021-05209-2 (PMC8816513; doi:10.1007/s00467-021-05209-2)
Supplement: Supplementary file 2 — A higher resolution version of the Graphical abstract is available as Supplementary information (PPTX 43.8 kb) [file 467_2021_5209_MOESM2_ESM.pptx]

## Slide 1
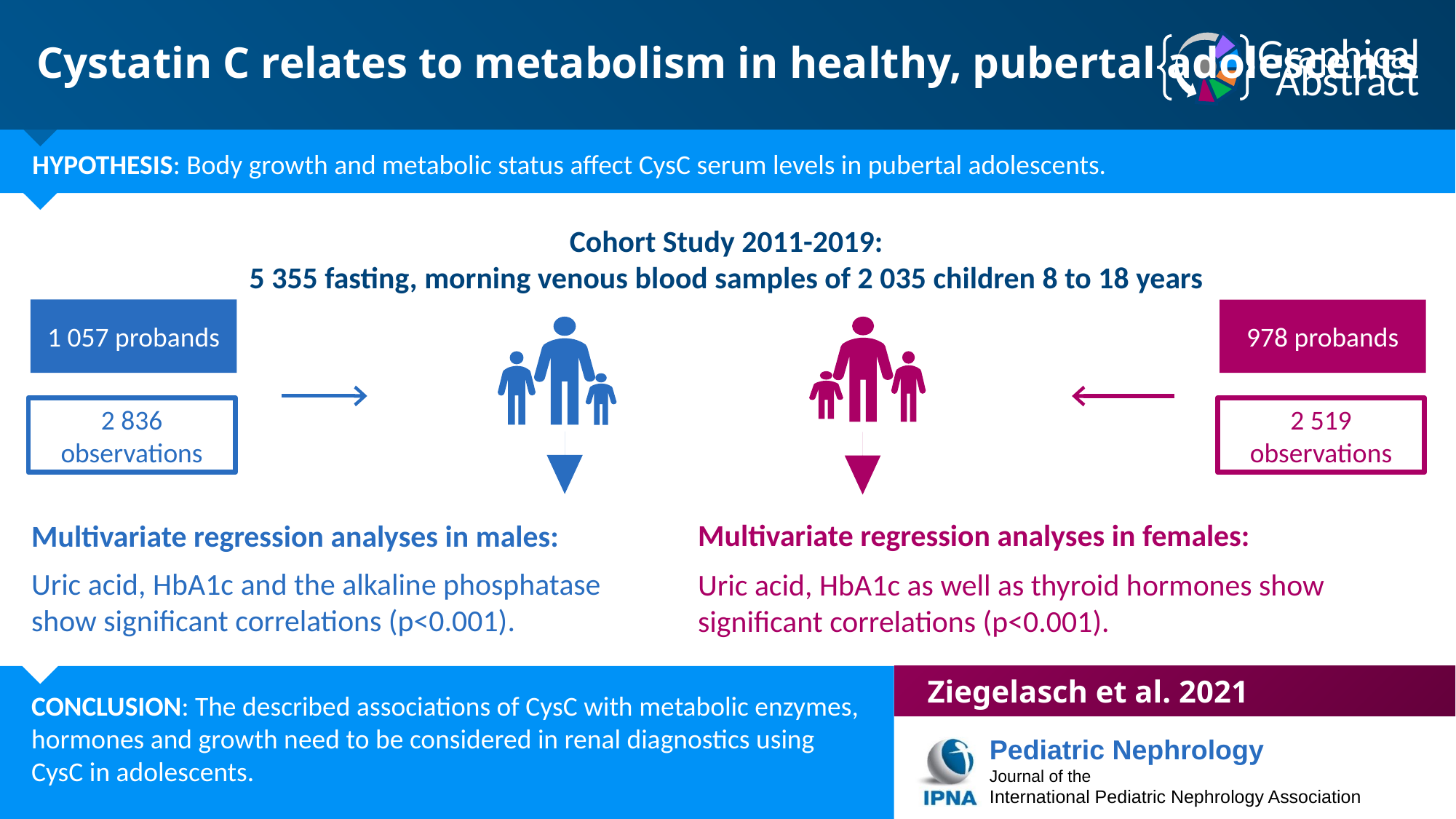

Cystatin C relates to metabolism in healthy, pubertal adolescents
HYPOTHESIS: Body growth and metabolic status affect CysC serum levels in pubertal adolescents.
Cohort Study 2011-2019:
5 355 fasting, morning venous blood samples of 2 035 children 8 to 18 years
1 057 probands
978 probands
2 836 observations
2 519 observations
Multivariate regression analyses in females:
Multivariate regression analyses in males:
Uric acid, HbA1c and the alkaline phosphatase show significant correlations (p<0.001).
Uric acid, HbA1c as well as thyroid hormones show significant correlations (p<0.001).
Ziegelasch et al. 2021
CONCLUSION: The described associations of CysC with metabolic enzymes, hormones and growth need to be considered in renal diagnostics using CysC in adolescents.
